# Momelotinib versus ruxolitinib in JAK inhibitor-naïve patients with myelofibrosis: an efficacy/safety analysis in the Japanese subgroup of the phase 3 randomized SIMPLIFY-1 trial

**DOI:** 10.1007/s12185-024-03822-z

**Published:** 2024-08-07

**Authors:** Kazuya Shimoda, Norio Komatsu, Itaru Matsumura, Kazuhiko Ikeda, Masayuki Hino, Michihiro Hidaka, Yoshinobu Maeda, Takeshi Kondo, Tomoaki Fujisaki, Keita Shoshi, Kyoichi Azuma, Ryuichi Fukushima, Jun Kawashima, Hiroshi Kosugi

**Affiliations:** 1 Hematology, Diabetes, and Endocrinology, University of Miyazaki, 5200 Kihara, Kiyotake, Miyazaki 889-1692 Japan; 2 Department of Hematology, Juntendo University School of Medicine, Tokyo, Japan; 3 Department of Hematology and Rheumatology, Kindai University Faculty of Medicine, Osaka-Sayama, Japan; 4 Department of Blood Transfusion and Transplantation Immunology, Fukushima Medical University School of Medicine, Fukushima, Japan; 5 Department of Hematology, Graduate School of Medicine, Osaka Metropolitan University, Osaka, Japan; 6 Department of Hematology, National Hospital Organization Kumamoto Medical Center, Kumamoto, Japan; 7 Department of Hematology, Oncology and Respiratory Medicine, Okayama University Graduate School of Medicine, Dentistry, and Pharmaceutical Sciences, Okayama, Japan; 8 Department of Hematology, Faculty of Medicine, Hokkaido University, Sapporo, Japan; 9 Blood Disorders Cente, Aiiku Hospital, Sapporo, Japan; 10 Department of Hematology, Matsuyama Red Cross Hospital, Matsuyama, Japan; 11 Clinical Development (Oncology), Japan Medical and Development, GlaxoSmithKline K.K., Tokyo, Japan; 12 Biostatistics, Japan Medical and Development, GlaxoSmithKline K.K., Tokyo, Japan; 13 Sierra Oncology, Inc., San Mateo, USA; 14 Department of Hematology, Ogaki Municipal Hospital, Ogaki, Japan

**Keywords:** Momelotinib, Phase 3, Myelofibrosis, Ruxolitinib, Japan

## Abstract

Momelotinib, an oral Janus kinase (JAK) 1/2 and activin A receptor type 1 inhibitor, improved symptoms, splenomegaly, and anemia in patients with myelofibrosis (MF). This sub-analysis of SIMPLIFY-1 evaluated the efficacy and safety of momelotinib versus ruxolitinib in Japanese patients with JAK inhibitor (JAKi)-naïve MF. Patients were randomized 1:1 to receive momelotinib 200 mg once daily or ruxolitinib 20 mg twice daily (or modified based on label) for 24 weeks, after which patients could receive open-label momelotinib. The primary endpoint was splenic response rate (SRR; ≥ 35% reduction in spleen volume) at 24 weeks; main secondary endpoints were total symptom score (TSS) response (≥ 50% reduction) and transfusion independence (TI) rates. Fifteen Japanese patients (momelotinib, n = 6; ruxolitinib, n = 9) were enrolled; all completed treatment. At Week 24, SRR was 50.0% with momelotinib and 44.4% with ruxolitinib. TSS response rates were 33.3% and 0%, and TI rates were 83.3% and 44.4%. Any-grade treatment-related adverse event (TRAE) rates were 83.3% with momelotinib and 88.9% with ruxolitinib. Grade 3/4 TRAE rates were 0% and 55.6%, with specific events being anemia (55.6%) and vertigo (11.1%) with ruxolitinib. Momelotinib was well tolerated, improved spleen and symptom responses, and reduced transfusion requirements in Japanese patients with JAKi-naïve MF.

**Supplementary Information:**

The online version contains supplementary material available at 10.1007/s12185-024-03822-z.

## Introduction

Myelofibrosis (MF) is a myeloproliferative neoplasm that manifests as bone marrow fibrosis, splenomegaly, cytopenias, such as anemia and thrombocytopenia, and constitutional symptoms, such as fatigue, fever, and night sweats [1]. These symptoms can profoundly affect quality of life and survival in patients with MF. The disorder can develop spontaneously, known as primary MF (PMF); or it can develop in people with polycythemia vera or essential thrombocythemia, known as secondary MF [1]. The worldwide annual incidence and prevalence of PMF stands at 0.44–0.52 and 1.76–4.05 per 100,000 people, respectively [2–4].

The underlying pathogenesis of MF has been extensively studied. Most patients with MF have mutations in *JAK2*, *CALR* or *MPL* genes, leading to the upregulation of Janus kinase (JAK)-signal transducer and activator of transcription (STAT) signaling [1, 5, 6]. As a result, there is an elevation of cytokine levels and growth factors, inflammation, cell proliferation and differentiation, resulting in the clinical manifestations of MF. Ruxolitinib was the first JAK 1/2 tyrosine kinase inhibitor approved for the treatment of MF, where it was shown to reduce splenomegaly and improved debilitating MF-related symptoms [7]. Studies in Japanese patients with MF have shown that ruxolitinib is associated with reduced spleen volume, and improvements to MF-related symptoms and quality of life [8–10]. However, ruxolitinib is also associated with dose-dependent anemia and thrombocytopenia, which is managed with dose reduction, interruption of treatment, or transfusions, but can lead to eventual discontinuation of treatment [7, 11].

Momelotinib, an oral JAK 1/2 and activin A receptor type 1 (ACVR1) inhibitor, has shown clinical activity on symptoms, spleen, and anemia of patients with MF [12–14]. Inhibition of the hyperactivated ACVR1 signaling pathway decreases hepcidin production, which consequently increases circulating iron and stimulates erythropoiesis, leading to improvement in iron-restricted anemia [15]. Momelotinib has been assessed in three randomized phase 3 studies (MOMENTUM, SIMPLIFY-1, and SIMPLIFY-2) and demonstrated improvements in symptoms, spleen size and anemia in patients with MF [12–14]. SIMPLIFY-1, a multicenter randomized double-blind phase 3 clinical trial, evaluated the efficacy and safety of momelotinib versus ruxolitinib in JAK inhibitor (JAKi)-naïve patients with MF [12]. The study met its primary endpoint of ≥ 35% reduction in spleen volume at 24 weeks and momelotinib was found to be noninferior to ruxolitinib for spleen response at Week 24 [12]. Although the secondary endpoint of noninferiority for a 50% reduction in total symptom score (TSS) at 24 weeks was not met, momelotinib was associated with increased transfusion independence and reduced transfusion burden compared with ruxolitinib [12]. Furthermore, momelotinib was associated with fewer adverse events (AEs) of anemia (13.6% vs 38.0%) and thrombocytopenia (18.7% vs 29.2%) compared with ruxolitinib [12].

To ensure that momelotinib is safe, efficacious, and applicable to diverse patient populations, a Japanese subgroup analysis of SIMPLIFY-1 was conducted.

## Materials and methods

### Study design, patient eligibility, stratification, and treatment

The methodology for SIMPLIFY-1 has been published previously [12]. In brief, SIMPLIFY-1 (NCT01969838) was an international, multicenter, double-blind, randomized phase 3 trial examining the efficacy and safety of momelotinib versus ruxolitinib in JAKi-naïve patients with PMF or post-polycythemia vera or post-essential thrombocythemia myelofibrosis (post-PV/ET MF).

This sub-analysis included Japanese patients treated at nine study centers in Japan. SIMPLIFY-1 enrolled patients aged ≥ 18 years old with palpable splenomegaly ≥ 5 cm below the left costal margin and a confirmed diagnosis of PMF or post-PV/ET MF in accordance with the World Health Organization (for PMF) or International Working Group for Myelofibrosis Research and Treatment criteria (for post-PV/ET MF) [16, 17]. Patients were classified by the International Prognostic Scoring System (IPSS) as high-risk, intermediate-2 risk, or intermediate-1 risk with symptomatic splenomegaly, hepatomegaly, anemia (hemoglobin [Hgb] < 10.0 g/dL), and/or unresponsive to non-JAKi therapy. Key inclusion criteria were: acceptable laboratory assessments within 14 days prior to the first dose of study treatment, including absolute neutrophil count ≥ 0.75 × 10^9^/L in the absence of growth factor therapy in the prior 7 days, platelet count ≥ 50 × 10^9^/L (≥ 100 × 10^9^/L if aspartate aminotransferase [AST] or alanine aminotransferase [ALT] ≥ 2 × upper limit of normal [ULN]) in the absence of platelet transfusion(s) or thrombopoietin mimetics in the prior 7 days, peripheral blood blasts < 10%, AST and ALT ≤ 3 × ULN (≤ 5 × ULN if liver is involved by extramedullary hematopoiesis as judged by the investigator or if related to iron chelator therapy that was started within the prior 60 days), creatinine clearance ≥ 45 mL/min and direct bilirubin ≤ 2.0 × ULN; Eastern Cooperative Oncology Group (ECOG) performance status score ≤ 2; and a life expectancy of > 24 weeks. Key exclusion criteria included prior use of a JAKi; prior splenectomy; spleen irradiation within 3 months before the first dose of study treatment; eligibility for allogeneic bone marrow or stem cell transplantation; use of chemotherapy, immunomodulating therapy, biologic therapy, radiation therapy, or investigational therapy within one month before the first dose of study treatment; certain cancers (history or concurrent disease); peripheral neuropathy Grade ≥ 2; or uncontrolled intercurrent illness that would limit study compliance as judged by the treating physician.

After the screening period, the study had a 24-week double-blind treatment phase. Patients were randomized 1:1 to receive momelotinib (200 mg once daily) or ruxolitinib (20 mg twice a day; or modified based on label). Randomization was stratified by transfusion dependence (yes or no; defined as ≥ 4 units of red blood cell [RBC] transfusions or Hgb level < 8 g/dL in the 8 weeks before random assignment, excluding patients associated with clinically overt bleeding) and by platelet count (< 100 × 10^9^/L, ≥ 100 × 10^9^/L and ≤ 200 × 10^9^/L, or > 200 × 10^9^/L). After completion of the 24-week double-blind treatment phase, patients in either arm had the option to receive momelotinib for up to an additional 216 weeks during the open-label phase or discontinue treatment. Patients in the ruxolitinib group with splenic progression had the option to begin the open-label phase of the study or proceed to the follow-up phase.

The trial protocol was reviewed and approved by the relevant institutional review boards. The study was conducted in accordance with the principles of the Declaration of Helsinki (Ethical Principles for Medical Research Involving Human Subjects), and ICH Guideline for Good Clinical Practice.

### Endpoints

The primary endpoint was the splenic response rate (SRR) at Week 24, where SRR was defined as the proportion of patients who achieved ≥ 35% reduction in spleen volume from baseline as measured by magnetic resonance imaging or computed tomography. Spleen volume was assessed at baseline, Week 12 and Week 24 during the double-blind phase, and every 12 weeks from Week 36 during and through to the end of the open-label phase. Secondary endpoints included TSS response rate at Week 24, where TSS response rate was defined as the proportion of patients who achieved ≥ 50% reduction in TSS from baseline as measured by the modified Myeloproliferative Neoplasm Symptom Assessment Form TSS diary; RBC transfusion independence (TI) rate at Week 24, where TI rate was defined as the proportion of patients who were transfusion independent (absence of RBC transfusion and no Hgb level < 8 g/dL in the prior 12 weeks); RBC transfusion dependence (TD) rate at Week 24, where TD rate was defined as the proportion of patients who were transfusion dependent (≥ 4 units of RBC transfusion or Hgb level < 8 g/dL in the prior 8 weeks); and rate of RBC transfusion, which was the average number of RBC units per patient-month during the double-blind treatment (through Week 24).

### Statistical analysis

All patient-level characteristics and sub-analysis results are described and summarized by treatment arm. Descriptive summaries show sample size, mean, standard deviation, and 95% confidence intervals on the mean, median, minimum, and maximum for continuous variables and counts, percentages, as well as 95% CIs on the percentage for categorical variables. A descriptive approach was used to assess primary and secondary endpoints: SRR, TSS, TI and TD at Week 24 during the double-blind phase, and SRR and Hgb at Week 48 during the open-label phase. Safety variables are described and summarized, including AEs and treatment-emergent AEs (TEAE).

## Results

### Patient characteristics

**Fig. 1:**
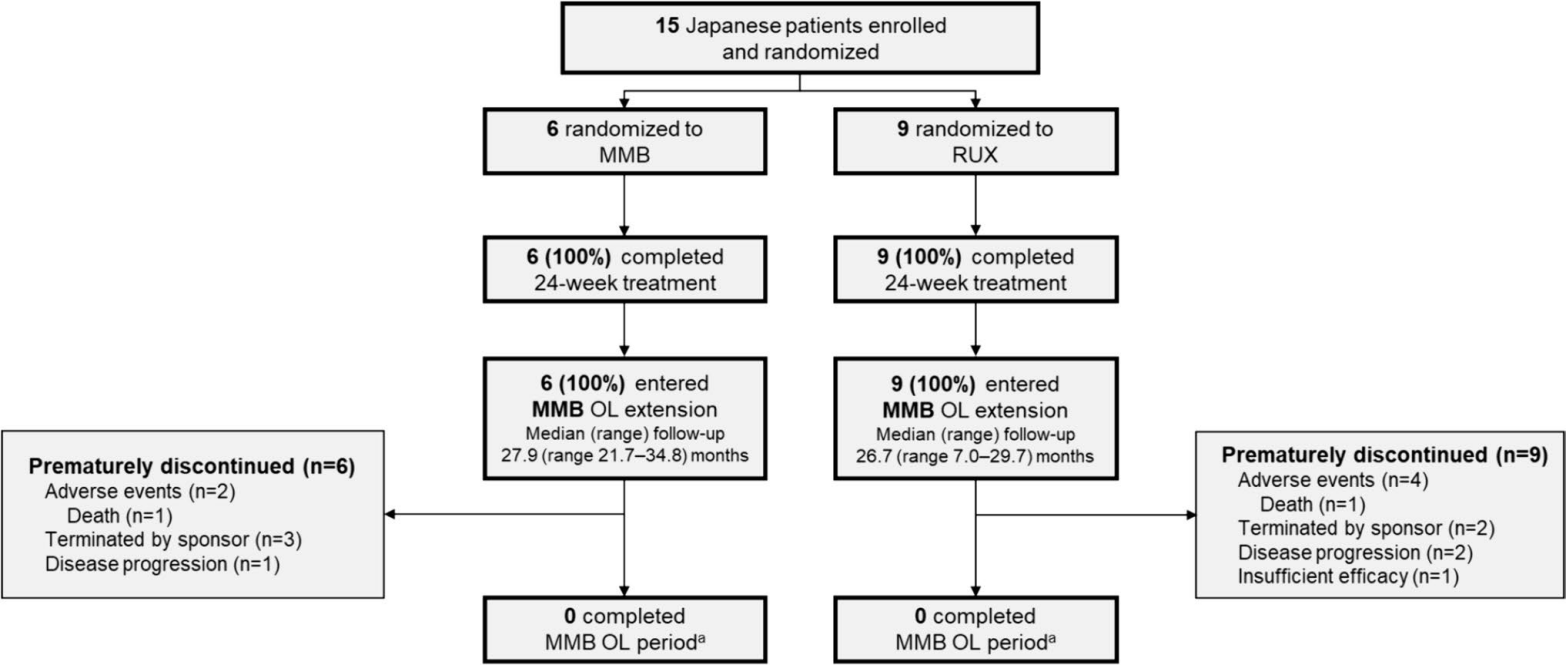
Patient disposition in the Japanese subpopulation. ^a^The study was prematurely discontinued by the sponsor closing the study, which was not related to momelotinib treatment [12]. MMB, momelotinib; OL, open-label; RUX, ruxolitinib

In this Japanese subpopulation analysis, 15 patients were randomized: six received momelotinib and nine received ruxolitinib. The median (range) dose was 200.0 (196.0–200.0) mg/day for momelotinib and 27.6 (10.0–40.0) mg/day for ruxolitinib. All patients completed the 24-week double-blind phase and all patients continued to the open-label phase (six from the momelotinib group continued momelotinib and nine from the ruxolitinib group switched to momelotinib) (Fig. 1). The study was prematurely terminated by the sponsor in order to transition to an extended access protocol (NCT03441113); however, none of the Japanese patients were transitioned. The median (range) duration of treatment exposure was 71.6 (18.3–114.9) weeks and 54.1 (4.3–83.0) weeks during the open-label phase for patients who continued with momelotinib and those who switched from ruxolitinib to momelotinib, respectively. Baseline patient and disease characteristics were relatively well balanced between the two treatment arms (Table 1).

**Table 1:** Baseline patient and disease characteristics

	Momelotinib (n = 6)	Ruxolitinib (n = 9)
Age, mean (SD)	63.5 (11.9)	65.4 (9.6)
< 65 years, n (%)	3 (50.0)	3 (33.3)
≥ 65 years, n (%)	3 (50.0)	6 (66.7)
Male, n (%)	5 (83.3)	5 (55.6)
Myelofibrosis subtype, n (%)		
Primary	3 (50.0)	4 (44.4)
Post-polycythemia vera	3 (50.0)	1 (11.1)
Post-essential thrombocythemia	0	4 (44.4)
IPSS risk category, n (%)		
Intermediate-1	1 (16.7)	1 (11.1)
Intermediate-2	5 (83.3)	1 (11.1)
High	0	7 (77.8)
JAK2V617F mutation, n (%)		
Previously tested	4 (66.7)	6 (66.7)
Positive	4 (66.7)	6 (66.7)
Negative	0	0
No	2 (33.3)	3 (33.3)
TSS score, median (range)	8.1 (5–47)	7.7 (0–24)
ECOG performance status, n (%)		
0	5 (83.3)	7 (77.8)
1	1 (16.7)	2 (22.2)
Hgb g/dL, mean (SD)	10.4 (2.9)	10.8 (2.7)
Hgb < 8 g/dL, n (%)	1 (16.7)	1 (11.1)
Hgb ≥ 8 g/dL, n (%)	5 (83.3)	8 (88.9)
Transfusion independent^a^ n (%)	3 (50.0)	8 (88.9)
Transfusion dependent^b^ n (%)	3 (50.0)	1 (11.1)
Central spleen volume cm^3^, mean (SD)	1581.7 (1309.6)	2060.0 (1472.1)
Platelet × 10^9^/L, mean (SD)	490.2 (402.5)	263.4 (108.2)
Absolute neutrophil count × 10^3^/μL, mean (SD)	9.7 (4.0)	18.8 (15.1)
White blood cell × 10^3^/μL, mean (SD)	12.4 (4.18)	25.3 (19.71)

ECOG, Eastern Cooperative Oncology Group; Hgb, hemoglobin; IPSS; International Prognostic Scoring System; RBC, red blood cell; SD, standard deviation; TSS, total symptom score

^a^Transfusion independence defined as not requiring RBC transfusion for ≥ 12 weeks, with Hgb levels ≥ 8 g/dL

^b^Transfusion dependence defined as requiring RBC transfusion ≥ 4 units or Hgb levels < 8 g/dL in the prior 8 weeks

### Efficacy

#### Double-blind phase

**Fig. 2:**
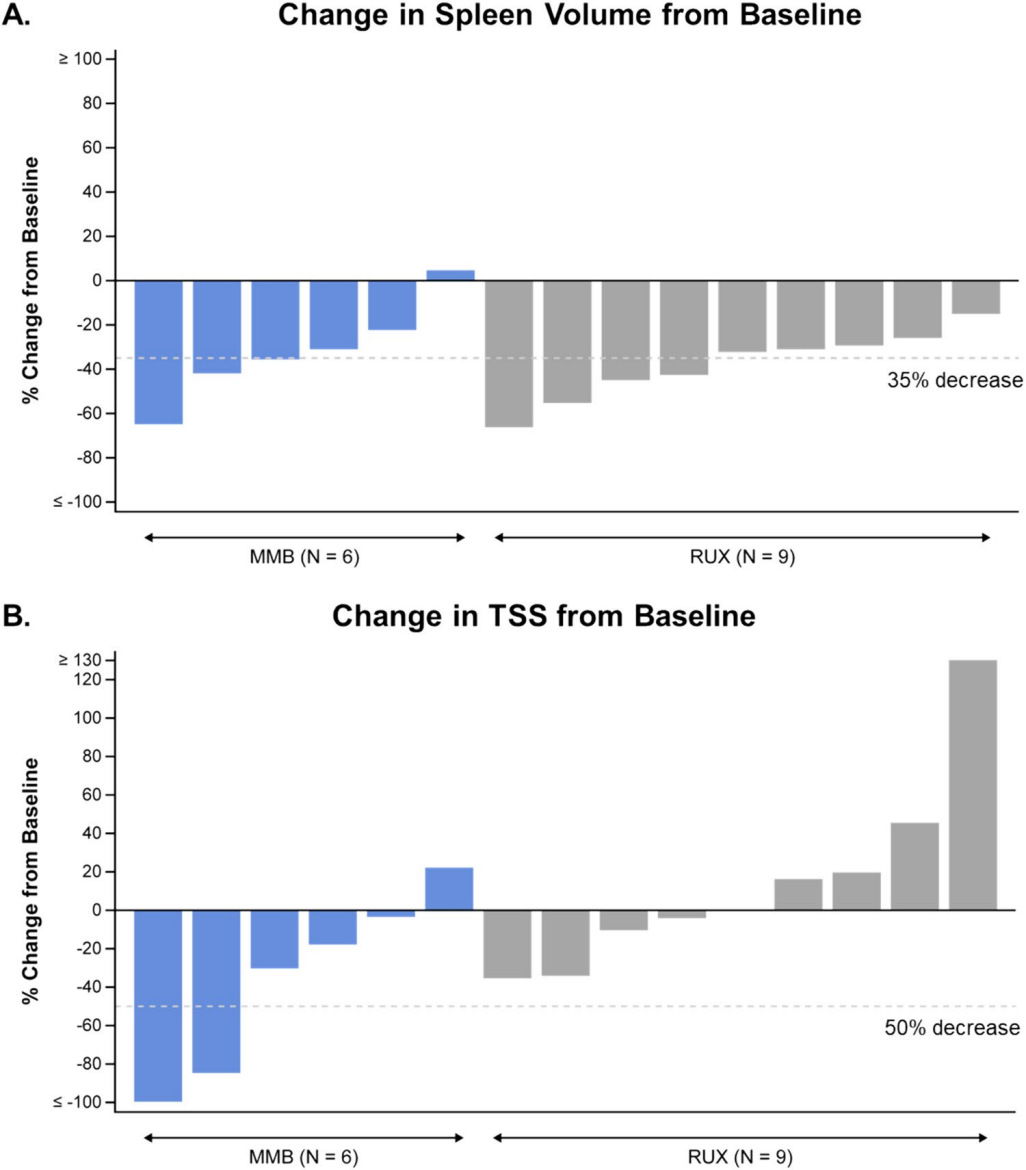
Percent change from baseline in **A** SRR and **B** TSS at Week 24. Dashed lines indicate the threshold response. MMB, momelotinib; RUX, ruxolitinib; SRR, splenic response rate; TSS, total symptom score

**Fig. 3:**
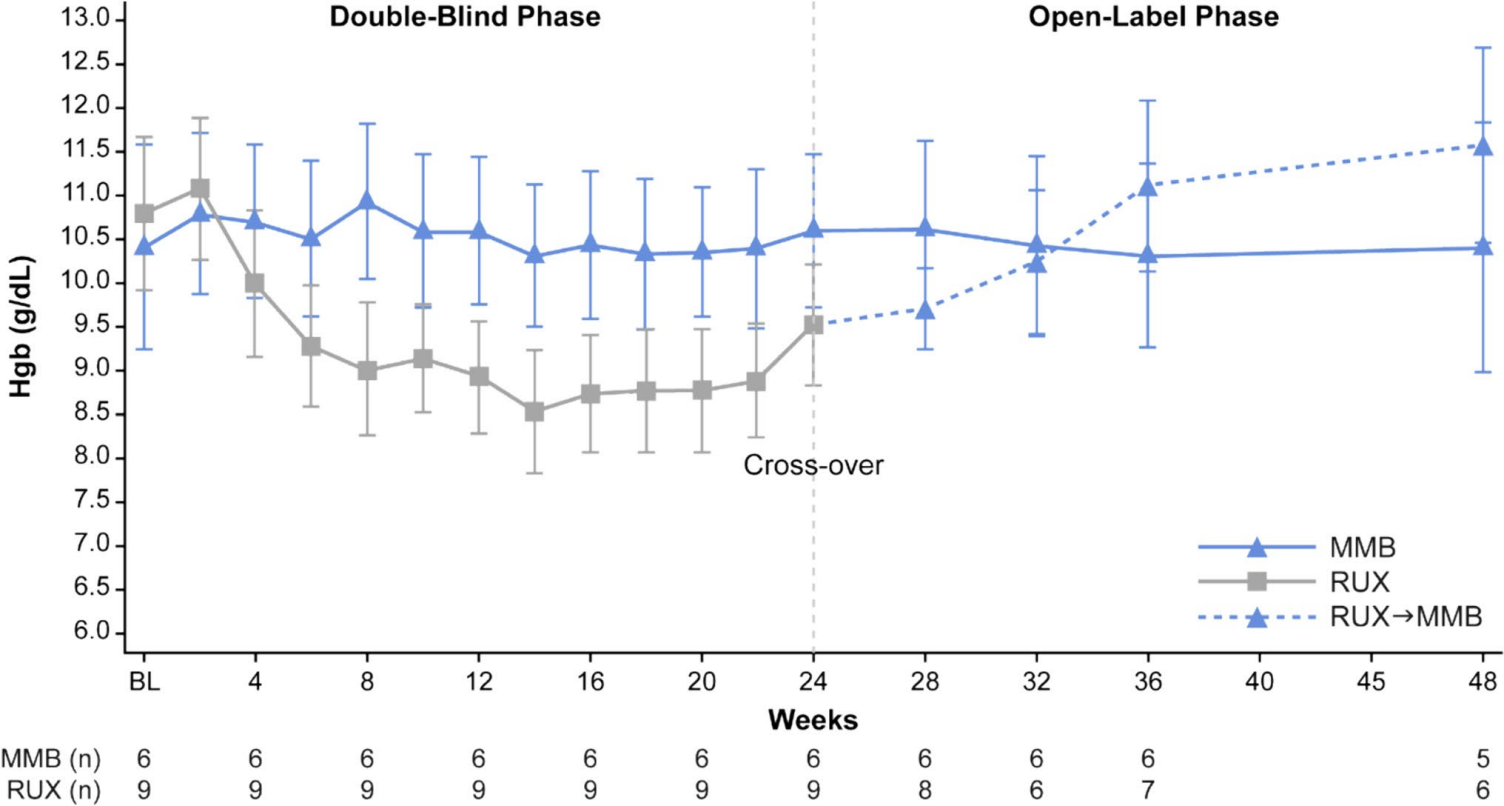
Mean hemoglobin levels during the double-blind and open-label phases to Week 48. Hgb, hemoglobin; MMB, momelotinib; RUX, ruxolitinib

All 15 patients who received momelotinib and ruxolitinib had assessments available for SRR at Week 24. SRR (≥ 35% reduction) was 50% (3/6) in patients who received momelotinib and 44.4% (4/9) in patients who received ruxolitinib (Table 2; Fig. 2A). All 15 patients who received momelotinib and ruxolitinib had assessments available for TSS at Week 24. More patients who received momelotinib had a ≥ 50% reduction in TSS compared with those who received ruxolitinib (33.3% [2/6] and 0% [0/9], respectively) (Table 2; Fig. 2B). More patients who received momelotinib were TI at Week 24 compared with the ruxolitinib group (83.3% [5/6] and 44.4% [4/9], respectively) and more patients who received ruxolitinib were TD at Week 24 compared with those receiving momelotinib (55.6% [5/9] and 16.7% [1/6], respectively) (Table 2; Supplementary Fig. 1). Compared to baseline, TI rate increased from 50.0 to 83.3% at Week 24 for momelotinib and decreased from 88.9 to 44.4% for ruxolitinib; TD rate decreased from 50.0 to 16.7% for momelotinib and increased from 11.1 to 55.6% for ruxolitinib. The median rate of RBC transfusion was 0 units/month for both the momelotinib and ruxolitinib groups. At baseline, the mean ± standard deviation (SD) Hgb concentration was 10.4 ± 2.9 g/dL in the momelotinib group and 10.8 ± 2.7 g/dL in the ruxolitinib group (Fig. 3). The mean maximum percent change in Hgb levels from baseline during the double-blind phase was 11.0% in the momelotinib group and 4.5% in the ruxolitinib group. At Week 24, mean ± SD Hgb was 10.6 ± 2.1 g/dL and 9.5 ± 2.1 g/dL for momelotinib and ruxolitinib, respectively.

**Table 2:** SRR, TSS, TI, and TD during double-blind phase (at Week 24)

	Momelotinib n = 6	Ruxolitinib n = 9
SRR		
Patients, n (%)	3 (50.0)	4 (44.4)
95% CI	(0.12, 0.88)	(0.14, 0.79)
TSS^a^		
Patients, n (%)	2 (33.3)	0
95% CI	(0.04, 0.78)	(0.00, 0.34)
RBC TI^b^		
Patients, n (%)	5 (83.3)	4 (44.4)
95% CI	(0.36, 1.00)	(0.14, 0.79)
RBC TD^c^		
Patients, n (%)	1 (16.7)	5 (55.6)
95% CI	(0.0042, 0.6412)	(0.2120, 0.8630)

CI, confidence interval; RBC, red blood cell; SRR, splenic response rate; TD, transfusion dependence; TI, transfusion independence; TSS, total symptom score

^a^TSS was defined as the proportion of patients who achieved ≥ 50% reduction in TSS from baseline

^b^Transfusion independence was defined as not requiring RBC transfusion for ≥ 12 weeks, with Hgb levels ≥ 8 g/dL

^c^Transfusion dependence defined as requiring RBC transfusion ≥ 4 units or Hgb levels < 8 g/dL in the prior 8 weeks

#### Open-label phase

At Week 48, all patients were eligible for assessment of SRR. A splenic response was reported in 50.0% (3/6) of patients treated with momelotinib and 55.6% (5/9) of patients who switched from ruxolitinib to momelotinib (Supplementary Table 1). SRR was maintained from Week 24 to Week 48 in patients who remained on momelotinib, while SRR increased from 44.4% (4/9) to 55.6% (5/9) in patients who switched from ruxolitinib to momelotinib. Of the four patients who achieved a splenic response at Week 24 in the ruxolitinib group, three patients had Week 48 data and all three maintained a splenic response after switching to momelotinib. In patients who remained on momelotinib, mean ± SD Hgb levels were stable from Week 24 to Week 48 (10.4 ± 2.9 g/dL to 10.4 ± 3.2 g/dL) and mean ± SD Hgb levels increased from 9.5 ± 2.1 to 11.6 ± 2.8 g/dL from Week 24 to Week 48 in patients who switched from ruxolitinib to momelotinib (Fig. 3). The mean percent change in Hgb levels from Week 24 to Week 48 was 5.7% in the momelotinib group and 18.5% in the ruxolitinib group.

### Safety

#### Double-blind phase

During the 24-week double-blind phase, TEAEs were reported in 83.3% (5/6) of patients in the momelotinib arm and 88.9% (8/9) in the ruxolitinib arm (Table 3). No patients experienced a TEAE leading to death in either the momelotinib or ruxolitinib groups. Common any-grade TEAEs were thrombocytopenia (16.7%, 1/6), hyperuricemia (16.7%, 1/6) and peripheral sensory neuropathy (16.7%, 1/6) in patients who received momelotinib, and anemia (66.7%, 6/9) and thrombocytopenia (33.3%, 3/9) in patients who received ruxolitinib. Grade 1/2 peripheral sensory neuropathy was reported in 16.7% (1/6) and 11.1% (1/9) in the momelotinib and ruxolitinib groups, respectively, and Grade 1/2 infection in 0% and 11.1% (1/9). Patients experienced dose reduction or temporary interruption due to TEAEs in 0% and 44.4% (4/9) of patients in the momelotinib and ruxolitinib groups, respectively. Grade ≥ 3 TEAEs were reported in 0% and 55.6% (5/9) of patients in the momelotinib and ruxolitinib groups, respectively. Specifically, Grade ≥ 3 anemia (55.6%, 5/9) and vertigo (11.1%, 1/9) were reported in the ruxolitinib group.

**Table 3:** Overall summary of TEAEs during randomized treatment and first 24 weeks in open-label phase

	Double-blind phase (Week 1–Week 24)			Open-label extension phase (Week 24–Week 48)		
	Momelotinib n = 6	Ruxolitinib n = 9	Total N = 15	Continuing momelotinib → momelotinib n = 6	Switch ruxolitinib → momelotinib n = 9	Total N = 15
TEAE, n (%)	5 (83.3)	8 (88.9)	13 (86.7)	4 (66.7)	9 (100.0)	13 (86.7)
Grade ≥ 3	0	5 (55.6)	5 (33.3)	1 (16.7)	3 (33.3)	4 (26.7)
Related to study drug	2 (33.3)	8 (88.9)	10 (66.7)	2 (33.3)	7 (77.8)	9 (60.0)
Related to study drug with Grade ≥ 3	0	5 (55.6)	5 (33.3)	0	2 (22.2)	2 (13.3)
Serious TEAE	0	1 (11.1)	1 (6.7)	1 (16.7)	1 (11.1)	2 (13.3)
Serious TEAE related to study drug	0	0	0	0	0	0
Non-fatal SAEs	0	1 (11.1)	1 (6.7)	1 (16.7)	1 (11.1)	2 (13.3)
TEAE leading to premature discontinuation of momelotinib/momelotinib-matched placebo	0	0	0	1 (16.7)	1 (11.1)	2 (13.4)
TEAE leading to premature discontinuation of ruxolitinib/ruxolitinib-matched placebo	0	0	0	–	–	–
TEAE leading to dose reduction or temporary interruption of momelotinib/momelotinib-matched placebo	0	2 (22.2)	2 (13.3)	1 (16.7)	2 (22.2)	3 (20.0)
TEAE leading to dose reduction or temporary interruption of ruxolitinib/ruxolitinib-matched placebo	0	4 (44.4)	4 (26.7)	–	–	–
Death	0	0	0	0	1 (11.1)^a^	1 (6.7)
Grade 3/4 TEAE, n (%)						
Anemia	0	5 (55.6)	5 (33.3)	1 (16.7)	0	1 (6.7)
Hyperviscosity syndrome	0	0	0	1 (16.7)	0	1 (6.7)
Thrombocytopenia	0	0	0	0	1 (11.1)	1 (6.7)
Vertigo	0	1 (11.1)	1 (6.7)	0	0	0
Dehydration	0	0	0	1 (16.7)	0	1 (6.7)
Acute myeloid leukemia	0	0	0	0	1 (11.1)	1 (6.7)
Skin and subcutaneous tissue disorders	0	0	0	0	1 (11.1)	1 (6.7)

^a^Due to acute myeloid leukemia. SAE, serious adverse event; TEAE, treatment-emergent adverse event

#### Open-label phase

In the open-label phase, TEAEs were reported in 66.7% (4/6) of patients who remained on momelotinib and 100% (9/9) of patients who switched from ruxolitinib to momelotinib. One patient who switched from ruxolitinib to momelotinib experienced a TEAE leading to death due to acute myeloid leukemia. Peripheral sensory neuropathy was reported in 0% and 11.1% (1/9) of the momelotinib and ruxolitinib to momelotinib groups, respectively. There was a dose reduction or temporary interruption in 16.7% (1/6) of patients treated with momelotinib and 22.2% (2/9) of patients who switched to momelotinib. There were two treatment discontinuations due to TEAEs: 16.7% (1/6) of patients who continued on momelotinib and 11.1% (1/9) of patients who switched to momelotinib. Grade ≥ 3 TEAEs were reported in 16.7% (1/6) of patients who remained on momelotinib and 33.3% (3/9) of patients who switched to momelotinib. Specifically, anemia, hyperviscosity syndrome, and dehydration were each reported by 16.7% (1/6) of patients in the momelotinib arm; thrombocytopenia, acute myeloid leukemia, and drug eruption were each reported by 11.1% (1/9) of patients who switched to momelotinib.

## Discussion

In Japanese patients with JAKi–naïve MF, 24 weeks of momelotinib improved spleen and symptom responses, and reduced transfusion requirements. Overall, momelotinib was well-tolerated and had an acceptable safety profile in Japanese patients. However, the study was prematurely discontinued by the sponsor closing the study, which was not related to momelotinib treatment.

In the SIMPLIFY-1 primary analysis [12], spleen volume at Week 24 was reduced by ≥ 35% from baseline in 26.5% (57/215) of patients who received momelotinib and 29.0% (63/217) of patients who received ruxolitinib; and momelotinib was noninferior to ruxolitinib. Here, the proportion of Japanese patients who reached this primary endpoint was consistent with the intent-to-treat (ITT) population of the primary SIMPLIFY-1 study (momelotinib, 50.0%; ruxolitinib, 44.4%). Assessment of the secondary endpoints in the primary SIMPLIFY-1 analysis demonstrated that TSS at Week 24 was reduced by ≥ 50% from baseline in 28.4% (60/211) of patients who received momelotinib and 42.2% (89/211) of patients who received ruxolitinib, indicating noninferiority was not met [12]. While the TSS response rate at Week 24 for Japanese patients treated with momelotinib was in line with ITT, none of the patients treated with ruxolitinib showed a TSS response. The lack of a TSS response in the ruxolitinib group was unexpected, especially as there was a splenic response in this group, and this observation may be driven by the low TSS scores at baseline. In the ITT population, TI at Week 24 was reported in 66.5% and 49.3% of patients who received momelotinib and ruxolitinib, respectively, while TD at Week 24 was reported in 30.2% and 40.1% of patients [12]. In Japanese patients, the TI rate at baseline was higher in the ruxolitinib group compared to the momelotinib group (88.9% vs 50.0%); however, by Week 24 the TI rate increased in the momelotinib group and decreased in the ruxolitinib group, suggesting that momelotinib improved transfusion requirements in Japanese patients.

Ruxolitinib, a JAK1/2 inhibitor, is associated with improved splenomegaly and myelofibrosis-associated symptoms [7]. Here, SRR findings for ruxolitinib differ to previously reported studies in Japanese patients [8, 10]. In this sub-analysis the SRR and TSS for Japanese patients in the ruxolitinib group was 44.4% and 0%, respectively, while Komatsu et al*.* and Oritani et al*.* reported SRRs of 26% and 33%, respectively, and TSS response rates of 75% and 56% for Japanese patients treated with ruxolitinib [8, 10]. This suggests that the TSS response rate reported in this sub-analysis may be unusual; however, this may be due to differences in the proportion of patients in the IPSS high-risk category. In this sub-analysis, 77.8% of patients were in the high-risk category, while there were 54.9% patients in the high-risk category in Komatsu et al*.* [8]. Whether or not this contributes to the TSS response rate difference observed between studies requires further examination in studies with larger patient cohorts.

The pattern of Hgb levels throughout the double-blind and open-label phases was assessed in the primary SIMPLIFY-1 study [12]. Hgb levels in the momelotinib group increased from baseline at the first assessment point and remained stable from Week 2 to Week 24 and beyond during the open-label phase [18]. In the ITT population, Hgb levels in the ruxolitinib group were lower than the momelotinib group throughout the double-blind phase; however, Hgb levels increased in patients who switched from ruxolitinib to momelotinib [18]. In Japanese patients, mean Hgb levels during the double-blind phase were stable in the momelotinib group and followed a similar pattern to the ITT population. Similarly, during the double-blind phase, Hgb levels in the ruxolitinib group were lower than the momelotinib group, but the increase in Hgb levels once Japanese patients switched from ruxolitinib to momelotinib was greater than the levels reported in the ITT population. These findings demonstrated that Hgb levels in Japanese patients who received ruxolitinib improved after switching to open-label momelotinib, consistent with the trend seen in the MOMENTUM study of JAKi-experienced patients switching from danazol to momelotinib [13]. It is important to note that an increase in mean Hgb levels in the ruxolitinib group can be observed as early as Week 16 (before crossing over to momelotinib), which suggests that long-term treatment with ruxolitinib may also improve Hgb levels. This trend has been previously observed in the COMFORT-1 study, where mean Hgb levels decreased to approximately 9.5 g/dL in patients treated with ruxolitinib in the first 8–12 weeks before increasing to approximately 10.1 g/dL and remaining stable until the end of the study [19]. Despite this possibility, it is clear that Hgb levels are consistently higher in the momelotinib group than in the ruxolitinib group. This finding is due, in part, to the differentiated mechanism of action of momelotinib as an ACVR1 inhibitor, which has a beneficial effect on anemia [15]. Furthermore, the median rates of RBC transfusion in the momelotinib and ruxolitinib groups were similar in the Japanese sub-analysis and the primary SIMPLIFY-1 study, both favoring the momelotinib group [12].

Similar to the ITT population, baseline patient characteristics between the treatment groups in the Japanese analysis were relatively well balanced. It should be noted that most Japanese patients in the ruxolitinib group were IPSS high risk, while most patients in the momelotinib group were intermediate-2; however, this may be explained by differences in age and white blood cell (WBC) count. Specifically, age ≥ 65 years and WBC count > 25 × 10^9^/L are used in the IPSS risk classification; thus, the greater proportion of patients categorized as high risk in the ruxolitinib group compared with the momelotinib group may be due to the proportion of patients aged > 65 years (mean age 65.4 years vs 63.5 years) and WBC > 25 × 10^9^/L (mean WBC 25.3 × 10^9^/L vs 12.4 × 10^9^/L) [20].

In our analysis, Japanese patients who received momelotinib did not report dose reduction/discontinuation, Grade ≥ 3 TEAEs, or significant AEs in the double-blind phase. Across the double-blind phase, the most common Grade ≥ 3 TEAE was anemia, all of which was reported in the ruxolitinib group (55.6%, 5/9). Furthermore, it should be noted that JAK/STAT pathway inhibitors have been associated with neurotoxicity, including dizziness and peripheral neuropathy, with momelotinib being linked to peripheral neuropathy [21, 22]. Additionally, JAK inhibition is also associated with an increased risk of infection [5, 23]. Here, no Grade 3/4 peripheral neuropathy nor infections were reported in either the momelotinib or ruxolitinib treatment arms during the double-blind or open-label phases. However, one patient who switched from ruxolitinib to momelotinib during the open-label phase died due to acute myeloid leukemia; the investigator reported that the death was not related to treatment.

This sub-analysis is not without its limitations. Firstly, we have approached this Japanese sub-analysis with a descriptive approach to assess efficacy and safety. Secondly, this sub-analysis included a small sample size; however, we believe it is important to analyze and publish this data to provide some insight into the efficacy and safety of momelotinib in JAK inhibitor-naïve Japanese patients with myelofibrosis. These data may also be useful for patients with myelofibrosis after discontinuation of ruxolitinib as they may help to determine future treatment options. Future real-world studies in a larger Japanese population will be required to further explore the efficacy and safety of momelotinib.

## Conclusion

In Japanese patients with JAK inhibitor naïve MF, both momelotinib and ruxolitinib were safe and well tolerated; however, compared with ruxolitinib, momelotinib demonstrated more clinically meaningful and durable spleen, symptoms and anemia-related responses, including in patients who crossed over from ruxolitinib.

### Supplementary Information

Below is the link to the electronic supplementary material.

Supplementary file1 (DOCX 17 KB)

## Data Availability

For requests for access to anonymized subject level data, please contact corresponding author.
