# Supplementary material for: Momelotinib versus ruxolitinib in JAK inhibitor-naïve patients with myelofibrosis: an efficacy/safety analysis in the Japanese subgroup of the phase 3 randomized SIMPLIFY-1 trial
Source: Int J Hematol. 2024 Aug 7;120(3):314–24. doi: 10.1007/s12185-024-03822-z (PMC11362197; doi:10.1007/s12185-024-03822-z)
Supplement: Supplementary file 1 — Supplementary file1 (DOCX 17 KB) [file 12185_2024_3822_MOESM1_ESM.docx]

**Title:** Momelotinib versus ruxolitinib in JAK inhibitor-naïve patients with myelofibrosis: an efficacy/safety analysis in the Japanese subgroup of the phase 3 randomized SIMPLIFY-1 trial

Supplementary Table 1. Splenic response rate from Week 36 to Week 108 of open-label phase

|  | **Momelotinib (n=6)** | **Ruxolitinib→ Momelotinib**  **(n=9)** |
| --- | --- | --- |
| Week 36 |  |  |
| Splenic response, n (%) | 2 (33.3) | 5 (55.6) |
| Change from baseline (%), mean (SD) | -28.1 (30.2) | -40.5 (15.1) |
| Week 48 |  |  |
| Splenic response, n (%) | 3 (50.0) | 5 (55.6) |
| Change from baseline (%), mean (SD) | -34.3 (29.8) | -43.3 (14.3) |
| Week 60 |  |  |
| Splenic response, n (%) | 3 (50.0) | 3 (33.3) |
| Change from baseline (%), mean (SD) | -29.1 (36.5) | -39.3 (26.0) |
| Week 72 |  |  |
| Splenic response, n (%) | 2 (33.3) | 4 (44.4) |
| Change from baseline (%), mean (SD) | -29.2 (45.1) | -39.0 (28.4) |
| Week 84 |  |  |
| Splenic response, n (%) | 2 (33.3) | 3 (33.3) |
| Change from baseline (%), mean (SD) | -23.9 (47.0) | -36.0 (40.7) |
| Week 96 |  |  |
| Splenic response, n (%) | 2 (33.3) | 1 (11.1) |
| Change from baseline (%), mean (SD) | -44.0 (20.6) | -12.9 (57.1) |
| Week 108 |  |  |
| Splenic response, n (%) | 1 (16.7) | 0 |
| Change from baseline (%), mean (SD) | -44.6 | - |

Supplementary Figure 1. Transfusion requirements during the double-blind phase


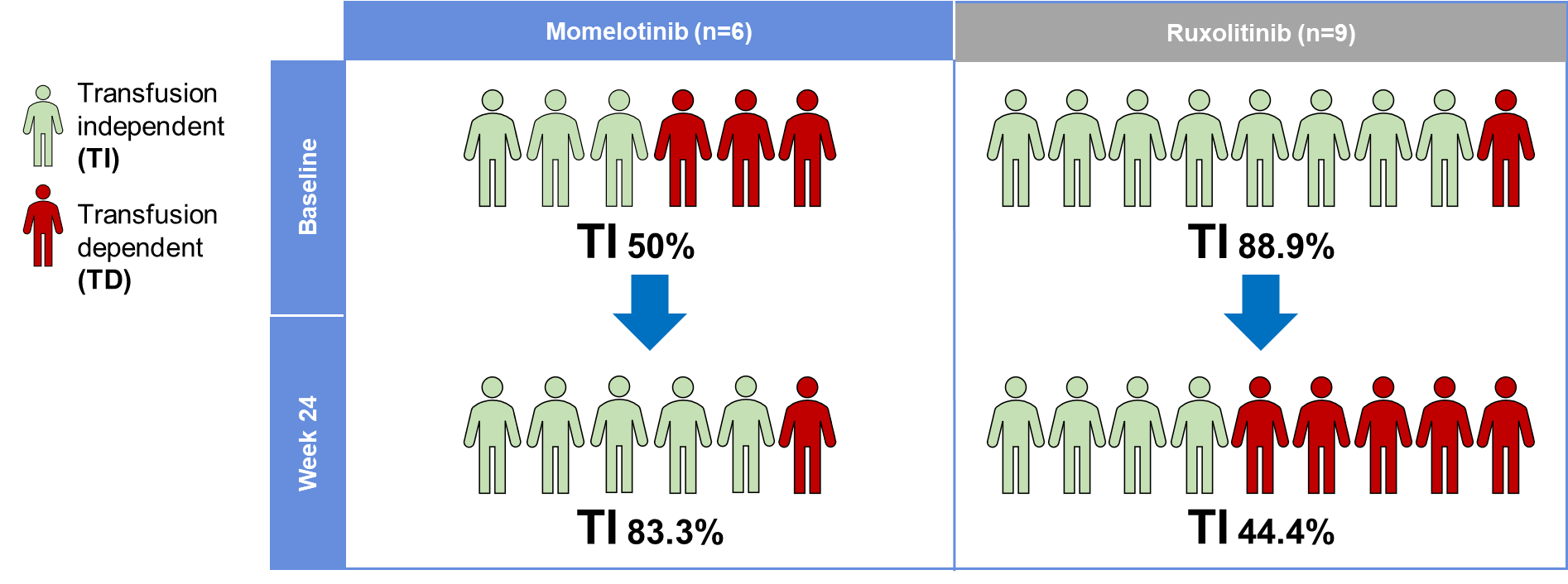


TD, transfusion dependent; TI, transfusion independent
